# A Case of Central Venous Sinus Thrombosis in a Young Woman

**DOI:** 10.5811/cpcem.2019.6.42988

**Published:** 2019-08-05

**Authors:** Liam F. Delahanty, Timothy G. Parker

**Affiliations:** * Naval Hospital Okinawa, Department of Emergency Medicine, Okinawa, Japan; † Flight Surgeon, HMH-466 “Wolfpack,” Camp Pendleton, California

## Abstract

Altered mental status is a common symptom in emergency department evaluations and may be present in as many as four to ten percent of patients.^1^ The etiology can be difficult to determine without significant evidence from laboratory, radiographic and physical examination. The diagnostic approach is largely driven by the provider’s clinical judgment based on the available history. Consequently, less-common diagnoses can be easily missed or delayed if a reasonable suspicion does not exist when considering possible causes. Cerebral venous sinus thrombosis (CVST) is one such uncommon, seldom-considered disease that carries a significant morbidity and mortality. Its clinical presentations vary and it disproportionally affects young to middle-aged individuals. Knowledge of the disease, particularly the risk factors, is key to making the diagnosis. We will discuss the case of a patient who presented with CVST and intraparenchymal hemorrhage in a resource-limited environment.

## INTRODUCTION

Cerebral venous sinus thrombosis (CVST) is a relatively rare yet clinically significant disease that can affect young patients in the prime of their lives. Its presentations can range from the subtle to the dramatic and profound. However, if identified in a timely manner, appropriate treatment results in full recovery in many cases.^2^ The current gold standard for diagnosis of CVST is magnetic resonance imaging venography (MRV), although computed tomography venography (CTV) was shown to have nearly comparable capability to identify CVST.^3^ This is important for settings in which MRV is not readily available in order to expedite disposition and initiation of anticoagulant therapy. Here we discuss the case of an otherwise healthy female who presented with intraparenchymal hemorrhage and altered mental status (AMS) as a result of CVST. With appropriate anticoagulation therapy, she made a full recovery.

## CASE REPORT

A 31-year-old female, with a past medical history significant for a right upper extremity deep venous thrombosis a decade prior, presented to the emergency department (ED) with a chief complaint of AMS. The history was obtained from the patient’s husband as her condition allowed only minimal participation in the history and physical exam. Symptoms had developed over the preceding 24 hours, beginning with a mild headache and progressing to stupor, aphasia, and finally urinary incontinence. The husband stated the symptoms began gradually, adding that the patient’s only medication was an oral, estrogen-containing contraceptive. She had been in her usual state of health prior to onset of symptoms in the prior 24 hours.

The patient was afebrile with vital signs in the normal range for her age. Her physical exam was notable for a well-nourished, well-developed young woman without apparent signs of distress. There were no focal abnormalities to her neurologic exam noted in the ED. There were no signs of trauma on her physical exam. Her Glasgow Coma Score (GCS) was 10; she was aroused only to painful stimuli and was not following commands. Her blood glucose was within normal limits and there was no response to empiric dose of intravenous (IV) naloxone. Her laboratory workup in the ED was unremarkable.

The patient was deemed stable enough to go to the radiology suite. A frontal lobe intraparenchymal hemorrhage with evidence of early trans-tentorial herniation was noted on non-contrast CT (Image). The differential diagnosis included an arterial-venous malformation, tumor, or a hemorrhagic conversion of a CVST.

In the ED she was treated with mannitol, IV dexamethasone, and levetiracetam for seizure prophylaxis. The on-call neurosurgeon was consulted to see the patient in the ED, and the decision was made to proceed to the operating room. The patient was admitted to the neurosurgical service and underwent a decompressive craniectomy. When she was stable postoperatively the diagnosis of a superior sagittal sinus thrombosis was made with MRV. This imaging was delayed 24 hours from time of presentation secondary to availability of specialized radiology technicians.

She was started on a heparin infusion postoperatively after the diagnosis was confirmed. Following surgery the patient was transferred to a major transfer receiving hospital for postoperative and rehabilitation care. The patient had an excellent recovery. She was discharged from the hospital functionally independent and able to return to work in her previous career field.

## DISCUSSION

CVST is a relatively rare but clinically significant disease. The overall incidence in the adult population is approximately 1.32 per 100,000-person years.^4^ Mortality attributed to CVST ranks between 5.5–18% in recent series. Those patients who receive a timely diagnosis have a good chance for recovery; between 57–86% of patients achieve complete functional recovery.^5^

CVST is slightly more common in women, particularly in the 20- to 35-year-old age group. This difference is likely secondary to hypercoagulability associated with pregnancy and oral contraceptive use.^6^ The mean age of presentation in several large studies was 38 years of age.^6^ Hypercoagulable states are likely the major, and may be the only, identifiable risk factor for CVST. A Dutch study found an age-adjusted odds ratio (OR) of 13 for oral contraceptive use and risk of CVST.^7^ Ten hereditary prothrombotic conditions such as Factor V Leiden, deficiency of proteins C and S, and antithrombin III may account for 10–15% of cases of CVST.^7^ The OR for women using oral contraceptives who also carry a diagnosis of a prothrombotic defect was calculated at 30.^7^

**CPC-EM Capsule**

What do we already know about this clinical entity?

*Central venous sinus thrombosis (CVST) is an uncommon, but clinically significant cause of headache that affects the majority of patients in early adulthood*.

What makes this presentation of disease reportable?

*Our patient manifested CVST as altered mental status on her index visit to the emergency department. This is an uncommon and potentially confounding presentation*.

What is the major learning point?

*Given a high index of suspicion, appropriate imaging modalities (magnetic resonance venography, computed tomography venography) will help assure timely diagnosis*.

How might this improve emergency medicine practice?

*Awareness of the varied presentations of CVST and the best imaging modalities for its diagnosis contributes to a general understanding of treatment options*.

Unlike in our case where AMS was the chief complaint, often a simple headache is the presenting symptom in 70–90% of cases.^3^ More dramatic presentations such as stroke-like symptoms including focal deficits such as hemiparesis and hemisensory disturbance, seizures, impairment of level of consciousness, and papilledema occur in one-third of cases.^3^ Intracerebral hemorrhage occurs in 35–39% of patients suffering CVST.^8^

The diagnosis of CVST can be elusive. As many as one in 18 cases of CVST are misdiagnosed at the index visit to the ED as measured by ED return visits.^9^ Classically MRV has been thought to be the gold standard for diagnosis; however, institutional and clinical constraints may make obtaining this study impossible. Magnetic resonance imaging (MRI) with gradient echo T2* susceptibility-weighted sequences with MRV has a higher sensitivity for detection of CVST and also has the advantage of demonstrating age-dependent signal features.^10^ However, it is not without its own limitations, particularly as a result of mimicry of sinus thrombosis by normal anatomic variants such as sinus atresia or hypoplasia, asymmetric sinus drainage, and filling defects as a result of arachnoid granulations or intra-sinus septa.^3^ In such cases, the inclusion of digital subtraction angiography (DSA) may be necessary to establish the diagnosis as well as to rule out dural arteriovenous fistulas and distal aneurysms prior to the initiation of anticoagulant therapy in cases where subarachnoid hemorrhage is also present.^10^

Non-contrast CT is near universal in its availability, which often makes CT the first imaging study for headaches if imaging is indicated. Unfortunately, changes indicative of CVST are only visible on approximately 30% of non-contrast CTs.^3^ One visible abnormality is the dense/filled delta sign, a hyperdensity in the posterior portion of the superior sagittal sinus. Other changes that may be apparent on contrast-enhanced CT include the “empty delta sign,” reflecting the opacification of collateral veins in the wall of the superior sagittal sinus, and the “cord sign,” a curvilinear hyperdensity over the cerebral cortex due to thrombosed cortical veins.^7^

When access to MRV is limited it is widely accepted that CTV is a reasonable alternative in the identification of CVST with sensitivity and specificity of 95% and 91%, respectively, when compared to DSA.^3^ Additionally, advantages of CTV include timely image acquisition, no contraindication to pacemaker or ferromagnetic hardware, increased image resolution, and fewer equivocal findings per some reports.^10^ It is also less invasive than diagnostic tests such as DSA. Conversely, it would be a less suitable alternative should the patient have iodine-contrast material allergies or poor renal function, limiting the ability to introduce contrast, while the intrinsic factor of radiation exposure also exists.^3^

Of note, lumbar puncture and cerebral spinal fluid (CSF) analysis is likely unhelpful in establishing a primary diagnosis of CVST. Abnormalities are nonspecific (increased opening pressure, increased red blood cell counts, increased protein content, and pleocytosis), occurring in 84% of cases without pathognomonic features.^2^ Rather, CSF analysis is likely helpful at narrowing the differential diagnosis for CVST by identifying clinical mimics such as meningitis and subarachnoid hemorrhage.

Once diagnosed, initial management in CVST is focused on correcting and managing serious complications such as increased intracranial pressure to prevent herniation, seizures, and stroke. As was the case with our patient, decompressive craniectomy may be required if left untreated or if mass effect resulting in a herniation syndrome occurs.^11^

Definitive treatment for CVST is generally focused on anticoagulation with thrombolysis. Surgical management is reserved for the most severe cases. While both unfractionated (UFH) and low molecular weight heparin (LMWH) have been demonstrated to be effective, UFH is more appropriate if surgical intervention may be needed and rapid reversal is desired.^11^–^13^ Meta-analysis of two randomized controlled trials with a total of 79 patients showed no statistically significant relative risk of death or dependence when patients were treated with IV UFH or subcutaneous nadroparin, a LMWH (relative risk 0.46, 95% confidence interval, 0.16 to 1.31).^3^ In one randomized controlled trial, anticoagulation showed treatment benefit. Moreover, no extension of hemorrhages present on initial evaluation or new hemorrhages were observed.^14^

Systemic thrombolysis, catheter-directed thrombolysis, and mechanical thrombectomy may be used for cases refractory to anticoagulation. Li G et al. treated 52 patients with severe CVST using mechanical thrombectomy combined with injecting urokinase via a catheter and reported 87% of these patients achieved complete recanalization.^15^

When CVST results in poor outcomes, they are often associated with risk factors such as central nervous system infection, malignancy, thrombosis of the deep venous system, intracranial hemorrhage, GCS score < 9, mental status disturbance, age > 37 years, and male sex.^3^ Recurrence, however, is rare at 2.8 %.^16^

## CONCLUSION

This case demonstrates the severity with which CVST may present and how outcomes can be favorable with timely diagnosis and treatment. If risk factors are present in the setting of atraumatic brain bleeds, providers should maintain a reasonable degree of suspicion for CVST. When appropriate, CTV may be used as an acceptable alternative to MRV if the latter is not readily available. Such practice will enable providers in rural and resource-limited facilities to provide care more efficiently for patients with this rare but potentially life-altering condition.

## Figures and Tables

**Image:**
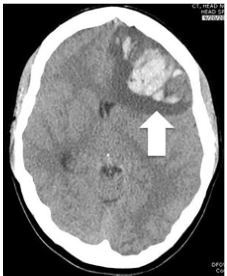
Non-contrast computed tomography of the patient demonstrating a frontal lobe intraparenchymal hemorrhage (arrow).
